# Improved Radiolytic Stability of a ^68^Ga-labelled Collagelin Analogue for the Imaging of Fibrosis

**DOI:** 10.3390/ph14100990

**Published:** 2021-09-28

**Authors:** Irina Velikyan, Ulrika Rosenström, Maria Rosestedt, Olof Eriksson, Gunnar Antoni

**Affiliations:** 1 Science for Life Laboratory, Department of Medicinal Chemistry, Uppsala University, SE-75183 Uppsala, Sweden; Maria.rosestedt@ilk.uu.se (M.R.); olof.eriksson@ilk.uu.se (O.E.); 2 PET-Centre, Centre for Medical Imaging, Uppsala University Hospital, SE-75185 Uppsala, Sweden; 3 Department of Medicinal Chemistry, Uppsala University, SE-75123 Uppsala, Sweden; Ulrika.rosenstrom@ilk.uu.se

**Keywords:** fibrosis, radiochemistry, collagen, collagelin, positron emission tomography, gallium-68, molecular imaging

## Abstract

There is an unmet medical need for non-invasive, sensitive, and quantitative methods for the assessment of fibrosis. Herein, an improved collagelin analogue labelled with gallium-68 for use with positron emission tomography (PET) is presented. A cyclic peptide, c[CPGRVNleHGLHLGDDEGPC], was synthesized by solid-phase peptide synthesis, conjugated to 2-(4,7-bis(2-(tert-butoxy)-2-oxoethyl)-1,4,7-triazonan-1-yl)acetic acid, and labelled with gallium-68. High performance liquid chromatography (HPLC) was used for the quality and stability assessment of the collagelin analogue. Non-specific organ distribution, blood clearance, and excretion rates were investigated in healthy mice and rats using ex vivo organ distribution analysis and dynamic in vivo PET/CT. Mice with carbon tetrachloride (CCl_4_) induced liver fibrosis were used for the investigation of specific binding via in vitro frozen section autoradiography, ex vivo organ distribution, and in vivo PET/CT. A non-decay corrected radiochemical yield (48 ± 6%) of [^68^Ga]Ga-NOTA-PEG_2_-c[CPGRVNleHGLHLGDDEGPC] ([^68^Ga]Ga-NO2A-[Nle^13^]-Col) with a radiochemical purity of 98 ± 2% was achieved without radical scavengers. The ^68^Ga-labelling was regioselective and stable at ambient temperature for at least 3 h. The autoradiography of the cryosections of fibrotic mouse liver tissue demonstrated a distinct heterogeneous radioactivity uptake that correlated with the fibrosis scores estimated after Sirius Red staining. The blood clearance and tissue washout from the [^68^Ga]Ga-NO2A-[Nle^13^]-Col was fast in both normal and diseased mice. Dosimetry investigation in rats indicated the possibility for 4–5 PET/CT examinations per year. Radiolytic stability of the collagelin analogue was achieved by the substitution of methionine with norleucine amino acid residue without a deterioration of its binding capability. [^68^Ga]Ga-NO2A-[Nle^13^]-Col demonstrated a safe dosimetry profile suitable for repeated scanning.

## 1. Introduction

The unmet medical need for the early, non-invasive, and quantitative diagnosis of fibrosis motivated the development of radiopharmaceuticals for nuclear molecular imaging using such technologies as single photon emission tomography (SPECT) and positron emission tomography (PET) [1,2,3,4,5,6,7]. The staging of the disease and monitoring of the disease progression are crucial for the efficiency of treatment planning. Drug development is another aspect of such an agent’s application wherein phase 0 clinical studies under a microdosing concept [8,9,10,11,12,13,14] can be conducted to investigate the pharmacokinetics of a drug of interest, thereby decreasing the cost of the development. Furthermore, with the advent of therapeutical agents [15,16,17] it is topical to develop a tool to investigate a drug’s action mechanism and to monitor the responses to a treatment accurately and quantitatively. It is not only important to detect the early stage of fibrosis but also to monitor the antifibrotic effect of therapeutic agents during treatment. The increasing number of clinical studies for the development of molecular imaging agents is a positive indication of progress in the field [7,18]. 

One of the targets for radioactive imaging agents is type I collagen which is the most abundant extracellular matrix protein [1,2,3,4,5,6]. Collagelin, a cyclic peptide identified from the bacterial peptide library, presumably binds selectively to the triple helix structure of collagen [1]. The aim of this study was to develop a PET tracer for the specific imaging of early active fibrosis via binding to monomer collagen. The uptake in bone, skin and other tissues rich in collagen cross-linked fibrils, and in diseased sites with established, mature collagen was expected to be low, consequently allowing a sufficient image contrast. Our initial cyclic peptide analogues demonstrated a rather low affinity in the micromolar range (Kd of 2.3 ± 0.8 μM and 2.1 ± 0.9 μM, respectively, for [^68^Ga]Ga-NO2A-Col and [^68^Ga]Ga-NODAGA-Col). However, due to the high micromolar concentration of collagen I during active fibrosis, the binding of the agent might not saturate the target or provide a sufficiently high radioactivity uptake. It was demonstrated that a peptide-based radioactive probe targeting collagen I was more sensitive to newly formed and less organized collagen [4]. A specific binding commonly requires strong affinity (low value of K_d_, Equation (1)) to provide a sufficiently high binding potential and sensitivity of the imaging scan. However, in the case of collagen found in abundance, the high number of binding sites (B_max_) compensate for a high value of K_d_ leading to a high binding potential and consequently a high imaging sensitivity:

(1) $$\mathrm{BP}\;\left(\mathrm{Binding}\;\mathrm{Potential}\right)=\frac{B_{\max}\;\left(\mathrm{receptor}\;\mathrm{density}\right)}{K_{d}\;\left(\mathrm{affinity}\right)}\;$$

**Figure 1:**
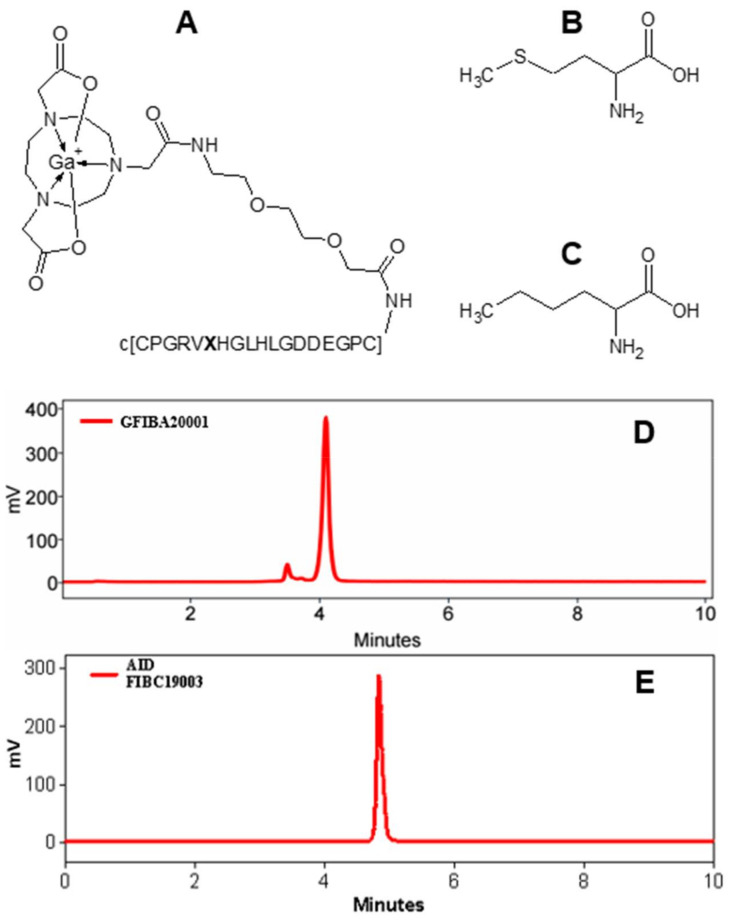
Chemical structures of [^68^Ga]Ga-NO2A-[**X**^13^]-Col (**A**), where X stands for either methionine (**B**) or norleucine (**C**). c[CPGRV**X**HGLHLGDDEGPC] is a cyclic peptide coupled to the chelator moiety via an ethylene glycol linker (EG_2_). (**D**) Radiochromatogram of [^68^Ga]Ga-NO2A-Col showing the by-products of radiolysis with a retention time below 4 min. (**E**) A typical radiochromatogram of [^68^Ga]Ga-NO2A-[Nle^13^]-Col demonstrating the absence of the by-products.

Thus, radiolabeled collagelin analogues are promising peptide scaffolds for PET imaging of fibrosis. However, the described analogues demonstrated a susceptibility to oxidative radiolysis presumably occurring at methionine amino acid residue. The radiolytic stability was improved in the presence of radical scavengers and the formation of the by-products was reduced, but the radiochemical purity of the analogues was not sufficiently high to assure a radiopharmaceutical grade quality (Figure 1A,B,D). To overcome this issue, we modified the collagelin by substituting the methionine amino acid residue with norleucine (Figure 1A–C), to decrease the sensitivity to oxidation. The aim of the present study was to further improve the characteristics of collagen analogues in terms of their radiolytic stability and to investigate the distribution of the novel oxidation-resistant analogue in a diseased mouse model.

## 2. Results

### 2.1. Chemistry and Radiochemistry

The new collagelin analogue was based on the [^68^Ga]Ga-NO2A-Col construct wherein the methionine was substituted with norleucine (Figure 1). 

The precursor, NO2A-[Nle^13^]-Col was synthesized with a chemical yield of 27% and purity of 97%, the Netto peptide content was estimated to be 70%. The resulting cyclic peptide (c[CPGRVNleHGLHLGDDEGPC]) conjugated to 2-(4,7-bis(2-(tert-butoxy)-2-oxoethyl)-1,4,7-triazonan-1-yl)acetic acid (NO2A) was labelled with gallium-68. The non-decay-corrected radiochemical yield was 48 ± 6% with a radiochemical purity of 98 ± 2%. The effective molar activity was 20 ± 7 MBq/nmol. No radiolabelled radiolysis by-products could be detected by radio-HPLC analysis of the [^68^Ga]Ga-NO2A-[Nle^13^]-Col (Figure 1E). The tracer stored at room temperature was stable for at least 3 h maintaining the same radiochemical purity as at the end of the synthesis (98 ± 2%, Figure 1E). 

### 2.2. In Vitro Binding Assay

**Figure 2:**
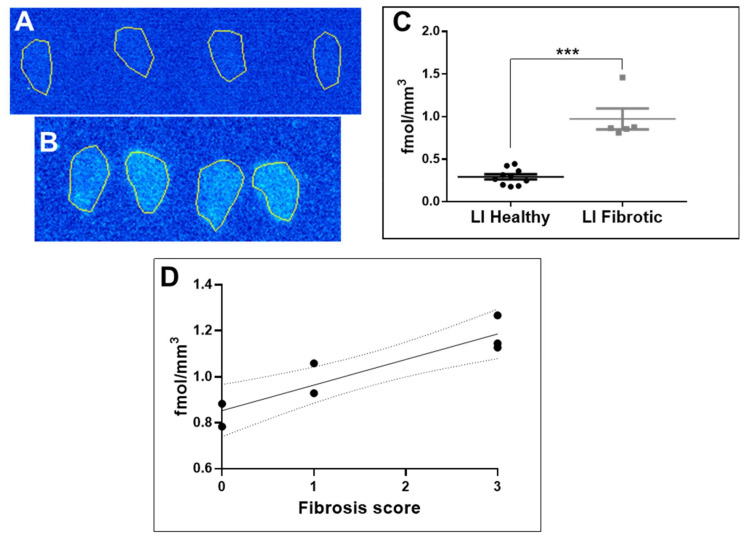
(**A**) Autoradiography of a frozen section of a normal mouse liver (a representative example). (**B**) Autoradiography of a frozen section of a liver of a mouse with induced liver fibrosis (a representative example). (**C**) Mann–Whitney test demonstrating the statistically significant difference (*p* = 0.0007; *** denotes values <0.001) between the uptake in the liver tissue of the healthy mice and mice with induced liver fibrosis. (**D**) Correlation of [^68^Ga]Ga-NO2A-[Nle13]-Col uptake in fibrotic mouse liver cryosections measured by autoradiography with a fibrosis score estimated by Sirius Red staining (R^2^ = 0.84; *p* = 0.0037).

The autoradiography of the cryosections of fibrotic mouse liver demonstrated a distinct heterogeneous uptake of the radioactivity (Figure 2B). The uptake in the diseased tissue (five independent measurements á 4–8 replicates) was higher than that in the healthy tissue (Figure 2A,B) with statistical significance (Figure 2C). The tracer binding in the liver cryosections investigated via autoradiography correlated with the grade of fibrosis estimated by the Sirius Red staining (Figure 2D). The background uptake was subtracted from the tissue uptake for the calculations of the binding site density (fmol/mm^3^).

### 2.3. Organ Distribution and Kinetics of [^68^Ga]Ga-NO2A-[Nle13]-Col in Mice: Healthy and with Induced Liver Fibrosis

**Figure 3:**
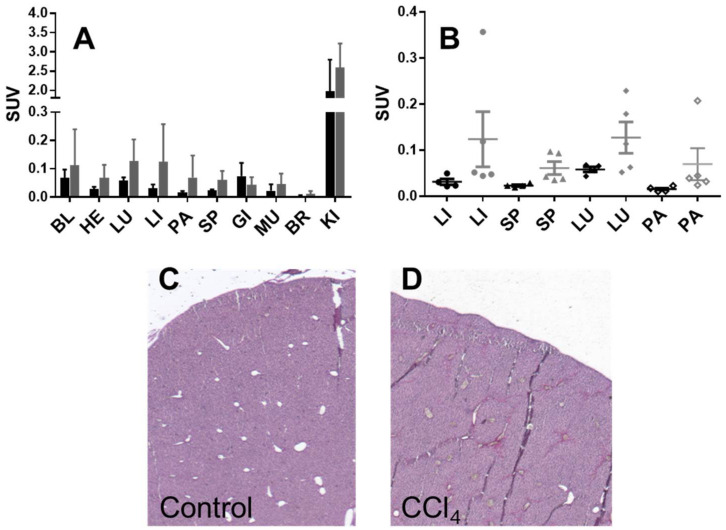
Organ distribution of ^68^Ga]Ga-NO2A-[Nle13]-Col in healthy mice (black bars (**A**) and points (**B**)) and mice with induced liver fibrosis (grey bars (**A**) and points (**B**)). BL: blood; HE: heart; LU: lungs; LI: liver; PA: pancreas; SP: spleen; MU: muscle; BM: red bone marrow; BR: brain; KI: kidneys. Representative Sirius Red staining images (4x magnification) of liver sections from mice with CCl_4_-induced fibrosis (**C**) and healthy controls (**D**).

Healthy mice (n = 4) and mice with induced liver fibrosis (n = 5) were administered with [^68^Ga]Ga-NO2A-[Nle^13^]-Col and the tracer organ distribution was studied after 60 min incubation (Table S1). The tracer uptake was assessed in 10 organs and presented as decay-corrected SUV values (Figure 3A). The uptake in all organs except for the kidney was below an SUV of one. Although the uptake was higher in the organs of mice with fibrosis as compared to the healthy mice, no statistically significant difference could be found in any organ including the liver, spleen, lung, and pancreas (Figure 3B). The kidney uptake indicated renal excretion. 

**Figure 4:**
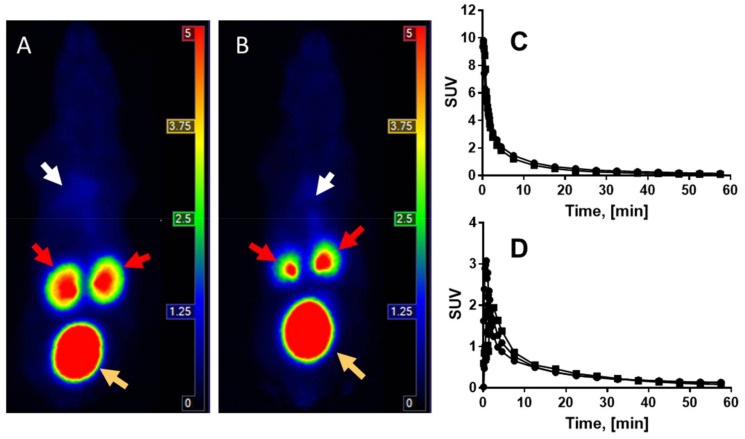
The representative animal PET/CT qualitative MIP images demonstrating whole body distribution of [^68^Ga]Ga-NO2A-[Nle13]-Col in healthy mouse (**A**) and a mouse with induced fibrosis (**B**) at 60 min post injection. White, red and orange arrows point respectively at the liver, kidneys, and urinary bladder. (**C**) Blood clearance from the heart of [^68^Ga]Ga-NO2A-[Nle13]-Col administered intravenously to healthy mice (R^2^ = 0.9533 (●)) and diseased mice (R^2^ = 0.994 (■)). (**D**) Washout from the liver of healthy (●) and diseased (■) mice.

Dynamic scanning during the 60 min incubation revealed fast blood clearance and washout from most of the organs for both healthy mice and mice with induced fibrosis (Figure 4A–D). The blood clearance kinetics were best described by a two-phase exponential with fast and slow phases (Figure 4C). The half-life values for the blood clearance were 0.53/4.8 min and 0.75/7.8 min, respectively, for healthy mice and diseased mice injected with ^68^Ga]Ga-NO2A-[Nle13]-Col.

### 2.4. Organ Distribution of [^68^Ga]Ga-NO2A-[Nle^13^]-Col in Healthy Rats and Human Dosimetry Calculation

The organ distribution of [^68^Ga]Ga-NO2A-[Nle13]-Col was studied in 15 male (weight: 363.3 ± 19.6 g; injected dose: 2.3 ± 0.8 MBq) and female (weight: 199.2 ± 5.5 g; injected dose: 1.4 ± 0.5 MBq) Sprague–Dawley rats at 5, 10, 30, 40, 60, and 120 min time points. Rapid clearance from the blood and wash-out from most of the organs with SUVs below 0.25 after 120 min was observed independently of gender. The highest SUV was around 12 and was shown by the kidneys indicating renal excretion with a decrease of 40–50% from 5 min to 120 min. The highest relative uptake in the kidneys was followed by the adrenal glands, bone marrow, and urinary bladder with SUVs below 0.25 at 120 min post administration. The remainder of the organs displayed SUVs below 0.1. 

**Figure 5:**
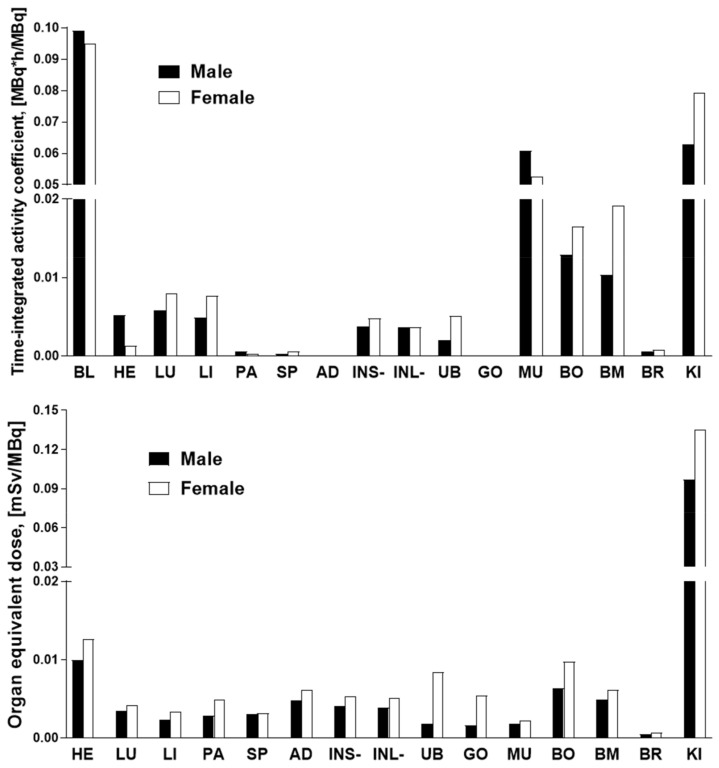
Graph showing the TIACs (**upper panel**) and estimated organ equivalent doses (mSv/MBq) (**lower panel**) of [^68^Ga] Ga-NO2A-[Nle13]-Col in human females and males extrapolated from rat ex vivo organ distribution data using OLINDA/EXM 1.1 software. BL: blood; HE: heart; LU: lungs; LI: liver; PA: pancreas; SP: spleen; AD: adrenals; INS+: small intestine with its content; INL: large intestine without content; UB: bladder; GO: gonads; MU: muscle; BO: bone; BM: red bone marrow; BR: brain; KI: kidneys.

**Figure 6:**
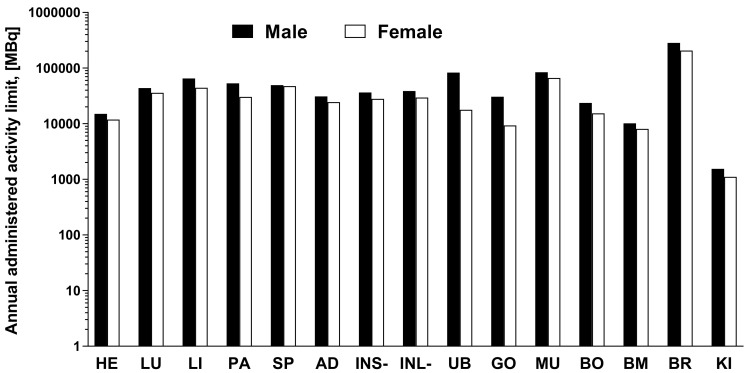
The maximum estimated administered dose (MBq) of [^68^Ga]Ga-NO2A-[Nle13]-Col calculated from respective organ effective dose (mSv/MBq) and the annual limit (mSv) for the organs in compliance with European Nuclear Society data. The y-axis is in logarithmic scale.

The data of the rat organ distribution was extrapolated to human organs assuming a similar biodistribution pattern [19]. The Time-integrated activity coefficient (TIAC), equal to the number of disintegrations (MBq-h/MBq) for each organ is presented in Figure 5. The highest TIACs were observed for the blood, kidney, muscle, bone, and bone marrow, in both genders. The effective dose and organ equivalent dose (mSv/MBq) were calculated using the OLINDA program with the TIACs as the input data. The tissue weighting factors recommended by the ICRP Publications 60 and 103 [20,21] were used for the calculations. This program gives consideration for the type of radiation. The remaining body TIAC was defined measuring the radioactivity in the remaining carcass, which refers to bulk radioactivity and is not related to the biodistribution or excretion of [^68^Ga]Ga-NO2A-[Nle13]-Col. Effective doses calculated by OLINDA, accounting for the radioactivity fraction in relation to the theoretical maximum TIAC, were 0.011 and 0.013 mSv/MBq, respectively, for male and female. The only dose limiting organ was the kidney. All other organs received a dose lower than 0.02 mSv/MBq (Figure 5). According to the European Nuclear Society, the annual limit for the kidney is 150 mSv. Thus, the annual injected dose of [^68^Ga]Ga-NO2A-[Nle13]-Col in MBq limited by kidney uptake would correspond to approximately 1100 MBq and 1500 MBq, respectively, for female and male. The estimated absorbed doses (mGy/MBq) for selected organs are summarized in Table 1, and dose limits of [^68^Ga]Ga-NO2A-[Nle13]-Col with respect to each organ are presented in Figure 6. 

## 3. Discussion

### 3.1. Chemistry and Radiochemistry

Our previous studies [2,3] demonstrated the susceptibility of collagelin analogues for oxidative radiolysis which was strongly influenced by an elevated temperature, the radioactivity amount, and the precursor concentration. It was necessary to suppress the radiolysis using radical scavengers and optimizing the heating temperature. The radiolysis of the peptide moiety was presumably attributed to the methionine residue, and in order to improve the radiolytic stability this residue was substituted with a more stable isosteric analogue, norleucine. Methionine has a long and non-polar side chain, which becomes polar upon oxidation leading to a more hydrophilic sequence with the possibility for an altered organ distribution pattern. The elimination of by-product formation by the substitution of Met to Nle is essential from the point of view of radiopharmaceutical purity and production robustness, desirably with the formation of a single radiochemical entity. The replacement of Met with Nle is considered conservative with a low probability for the functionality change of peptides and proteins, nevertheless, if the Met is crucial for the specific binding, then the anticipated targeting might be compromised. Moreover, not only the specific but also non-specific binding should be considered when exchanging amino acids since despite their structural similarity, the physicochemical properties of the side chains might differ and therefore influence the non-specific binding [22]. Nle is considered a close analogue of Met in terms of their physicochemical properties of composition, polarity, and molecular volume. However, being more hydrophobic, the Nle could alter the binding capacity of the peptide if the Met was essential for the binding process. Thus, the substitution of the oxidation-prone methionine with the non-oxidizable norleucine would on the one hand improve radiolytic stability while on the other hand demonstrate if the methionine were essential for the specific and non-specific binding ability of the agent. Another aim of this study was to conduct the in vivo and ex vivo characterization of the analogue in a fibrosis animal model.

One of the initially developed analogues (NO2A-Col) bearing a total positive charge at the gallium–chelate complex moiety demonstrated a lower uptake in the liver, spleen, and kidneys in healthy rodents [2,3] and was chosen for further modification. The substitution of Met with Nle improved the peptide stability against radiolysis, namely, no radiolabelled by-products could be detected (Figure 1E) even without radical scavengers, with a radiochemical purity of over 99%. Labelling of the NO2A-Col comprising methionine residue demonstrated a previous radioactivity incorporation of 35 ± 3% without and 95 ± 4% with radical scavengers [3]. The stability was further demonstrated by storing and monitoring the purity of the [^68^Ga]Ga-NO2A-Nle^13^-Col at an ambient temperature for at least 3 h. The absence of a chelating ability of the cyclic peptide moiety containing histidine and aspartate amino acid residues was earlier demonstrated for the NO2A-Col and NODAGA-Col [3] and was also confirmed for the NO2A-Nle^13^-Col.

### 3.2. Biological Characterization

The oxidation of the Met to methionine sulfoxide may reduce or even eliminate the binding affinity [23,24]. The replacement of Met with Nle may [24,25] or may not [26,27] influence the binding affinity. The binding ability of [^68^Ga]Ga-NO2A-[Nle^13^]-Col was studied in vitro via fibrotic mouse liver frozen section autoradiography, ex vivo and in vivo in mice with induced liver fibrosis.

The frozen section autoradiography performed on the liver tissue from healthy mice and mice with CCl_4_-induced fibrosis demonstrated a higher uptake in the diseased tissue as compared to the normal tissue with statistical significance (*p* = 0.0037, Figure 2A,B). The presence of excessive deposition of collagen was confirmed previously performing a Sirius Red histological assay on the adjacent tissue sections [6]. The uptake of [^68^Ga]Ga-NO2A-[Nle^13^]-Col in the fibrotic tissue linearly correlated (R^2^ = 0.84, Figure 2C) with the scoring of fibrosis, indicating a target density ranging between 0.7 and 1.3 fmol/mm^3^. The values are somewhat lower than those for [^68^Ga]Ga-NO2A-Col and [^68^Ga]Ga-NODAGA-Col (2.0 fmol/mm^3^) [3], as determined from frozen sections of dog heart affected by fibrosis, likely due to a more active fibrosis in the dog heart and an upregulated production of collagen. The linear correlation of [^68^Ga]Ga-NO2A-[Nle^13^]-Col uptake with the fibrosis scoring is an indication of the possibility for in vivo fibrosis staging, disease progression and treatment response monitoring.

Organ uptake of [^68^Ga]Ga-NO2A-[Nle^13^]-Col in healthy animals declined over time within 120 min for all organs, similar to [^68^Ga]Ga-NO2A-Col and without the retention in the liver and spleen that was observed previously for the [^68^Ga]Ga-NODAGA-Col. The blood clearance and wash-out rates from most of the organs was fast and independent of animal gender and was below an SUV of 0.1. At the one-hour post injection time point, the SUVs of all vital organs were below 0.2. The SUVs for most organs were somewhat higher for the female animals, however, the difference was not statistically significant. The blood clearance rate was slower for the mice with induced fibrosis as compared to the healthy mice. Nevertheless, in both cases it was sufficiently fast enough to provide a high contrast image 60 min post-injection matching the decay pace of the Ga-68 well. The low normal tissue uptake and retention was an indication of a low binding of the [^68^Ga]Ga-NO2A-[Nle^13^]-Col to the constitutive collagen. No statistically significant difference could be observed in the organ distribution of the three analogues in healthy mice and rats [2,3]. The highest uptake of [^68^Ga]Ga-NO2A-[Nle^13^]-Col was found for the kidneys with an SUV around 12, similar to the two previous analogues [2,3]. This was followed by the heart and bone, with the latter being physiologically rich in content of the type I collagen.

The variation of the organ uptake in the diseased animals was larger than in the healthy ones, most likely due to not only the variations in the fibrotic activity but also due to the unevenly scattered, fibrotic formations and consequent variations in tissue sampling. The in vivo imaging did not demonstrate a distinct uptake in the liver with induced fibrosis, however, ex vivo organ distribution showed a somewhat elevated uptake in the fibrotic liver as compared to the healthy one, although this was not statistically significant. The uptake difference between the normal and fibrotic tissue was more prominent in the in vitro experiments using the frozen liver sections. The absence of distinct tracer accumulation in the affected mice liver in vivo was presumably caused by low collagen production upregulation. The binding site density was presumably not sufficiently high enough to produce statistically sound radioactivity counts for the in vivo imaging in the mice. However, the aim of this research was to develop an imaging agent capable of detecting fibrosis during its early stage of development and thus the disease modelling was conducted using a relatively short-term (three weeks) repetitive administration of CCl_4_. The sensitivity of the techniques used for the biological characterization increases in the following order from in vivo micro-PET imaging to an ex vivo organ distribution to frozen section autoradiography. The detection of the specific binding effect improved with an increasing sensitivity of the detection methods. Further development will be aimed at improving the affinity of the agent to compensate for a relatively low binding site density.

The dosimetry study was conducted to estimate the potential radiotoxicity of the tracer to normal organs, especially such radiosensitive organs as the kidney and bone marrow, and to predict the possibility for multiple annual examinations. Ex vivo rat organ distribution data were extrapolated to humans with the assumption of a biodistribution similarity of the species and a radioactivity distribution homogeneity in the organs. The results of the dosimetry calculations demonstrated a similarity for the [^68^Ga]Ga-NO2A-Col and [^68^Ga]Ga-NODAGA-Col, though in contrast the [^68^Ga]Ga-NO2A-[Nle^13^]-Col showed a somewhat higher absorbed dose for the female as compared to the male. The limiting organ, as with the previous results, was the kidney with an effective dose of 0.097 mSv/MBq, similar to that for the [^68^Ga]Ga-NO2A-Col (0.102 mSv/MBq) and [^68^Ga]Ga-NODAGA-Col (0.097 mSv/MBq) for the male. While for the female it was somewhat higher (0.135 mSv/MBq) as compared to the [^68^Ga]Ga-NO2A-Col (0.103 mSv/MBq) and [^68^Ga]Ga-NODAGA-Col (0.111 mSv/MBq). The kidneys were followed by the heart with 0.010 (male) and 0.013 (female) mSv/MBq. The remaining organs received less than 0.009 mSv/MBq. As mentioned above, the difference in the absorbed doses of the liver and spleen for the [^68^Ga]Ga-NODAGA-Col and [^68^Ga]Ga-NO2A-Col drove the decision towards the latter for further development. The absorbed dose for the liver and spleen was lower for the [^68^Ga]Ga-NO2A-[Nle^13^]-Col than for the [^68^Ga]Ga-NO2A-Col (Table 1), further improving the radiation safety of the tracer. The absorbed dose for such radiation sensitive organs as the bone marrow was low allowing for the administration of 8000–10,000 MBq per year. The renal excretion pathway prevailed as in the case of the [^68^Ga]Ga-NODAGA-Col and [^68^Ga]Ga-NO2A-Col, leading to the kidney being identified as the limiting organ allowing for the intravenous administration of 1100 and 1500 MBq per year, respectively for females and males. The doses limited by the kidneys would correspond to approximately 5–15 PET examinations a year assuming an administration dose of 100–200 MBq. Further restriction is exerted by the limit of a maximum total effective dose of 10 mSv to healthy volunteers. The total effective dose of 0.011 (male) and 0.013 (female) mSv/MBq would result in 1.09 (male) and 1.34 (female) mSv per 100 MBq of injected radioactivity, allowing for 7–9 PET examinations. A PET/CT examination including CT (1 mSv) would give a total radiation dose of 2.09 (male) and 2.34 (female) mSv corresponding to 4–5 examinations per year. These estimations indicate that the effective dose is the limiting parameter in both genders. 

## 4. Materials and Methods

### 4.1. Peptide Synthesis

The synthesis of NO2A-[Nle^13^]-Col was conducted according to the previously published procedure [3] exchanging methionine amino acid with norleucine amino acid.

### 4.2. Radiolabeling and In Situ Stability Test

The ^68^Ge/^68^Ga generator (50 mCi, GalliaPharm, Eckert and Ziegler, Eurotope GmbH) eluate was fractionated providing 80–85% of the total ^68^Ga radioactivity in 3.0–3.5 mL of 0.1 M hydrochloric acid. The highest amount of radioactivity used in the synthesis was 900 ± 25 MBq. Thereafter the solution was buffered with a 250–350 µL of sodium acetate buffer (1M, pH 4.6) and 25-30 µL of sodium hydroxide (10M) providing a pH of 3.8–4.2 prior to the addition of 15–30 nanomoles (1 mM) of NOTA-PEG_2_-c[CPGRVNleHGLHLGDDEGPC]. The mixture was incubated in a 10 mL glass vial in a heating block at 75 °C for 10 min. The crude product solution was cooled down by mixing it with water (4 mL), and the product was purified by a solid-phase extraction using disposable cartridges (Oasis^®^ HLB). The retained product was eluted with 1 mL of 50% ethanol solution. The final product was formulated using either saline or phosphate buffered saline for subsequent biological assays.

A high-performance liquid chromatography system (HPLC, Agilent Technologies 1200 system) consisting of a 1290 pump, 1290 Vialsampler,1260 Variable Wavelength Detector (UV), and a radiation flow detector (Bioscan) coupled in series was used for the analysis of the crude and purified product. A reversed phase analytical column (Supelco, Discovery) was used for the separation of the analytes under the following conditions: A = 10 mM TFA in water; B = 70% acetonitrile (MeCN)/30% water/10mM TFA with UV-detection at 220 nm; linear gradient elution: 0–2 min at 20% B, 2–10 min from 20 to 100% B, 10–15 min 100% B; flow rate was 2.0 mL/min. Data acquisition and handling were performed using the OpenLAB Software Package. The HPLC method was validated in terms of radioactivity recovery from the analytical column wherein the analysis was performed, with and without the column under the exact same conditions collecting the effluent for the subsequent radioactivity measurement. The radioactivity of the collected samples was measured in an in-house built well-type NaI(Tl) scintillation counter and corrected for dead-time and for decay. The stability of the product at room temperature at reaction pH and at pH 7.4 in situ was monitored by UV-radio-HPLC at 2 and 3 h time points. The retention time for the radio-signal of [^68^Ga]Ga-NO2A-[Nle^13^]-Col was 4.1 ± 0.2 min..

### 4.3. In Vitro Binding Assay

The frozen tissues of liver from healthy mice and mice with induced hepatic fibrosis (3-week treatment) was sectioned (20 µm) using a cryostat microtome (Micron HM560, Walldorf, Germany). The sections were mounted on Menzel Super Frost plus glass slides, dried at room temperature (RT) and stored at −20 °C. The cryosections were pre-incubated in phosphate buffered saline (PBS, pH 7.4) containing 1% bovine serum albumin (BSA). For the determination of the total binding, the cryosections were incubated in a 10 mL buffer containing [^68^Ga]Ga-NO2A-[Nle^13^]-Col at a concentration of 2 µM at RT for 60 min. Thereafter, the sections were rinsed once for one min in ice-cold PBS containing 1% BSA and twice in ice-cold PBS. The cryosections were dried under a stream of warm air (37 °C, 7–10 min) and were exposed to phosphor imaging plates together with a reference solution (20 µL drop of incubation solution of known concentration) for approximately 10 min. The phosphor imaging plates were scanned using a Phosphorimager system (Amersham Typhoon IP, GE Health, North Richland Hills, TX, USA), and analyzed using ImageJ software (ImageJ 1.45S, NIH, Bethesda, MD, USA). The regions of interest (ROIs) were drawn on the images of liver sections, background, and reference. The retrieved median pixel intensity values of the sections and reference were corrected for those of the background. The median pixel intensity values were converted into moles using the reference of known concentration.

Liver sections from the same liver biopsies were previously stained with Sirius red to assess the grade of fibrosis [6].

### 4.4. Animals

The animals were kept in ventilated cages under a constant temperature (20 °C) and humidity (50%) in a 12 h light-dark cycle with free access to food and water. Local institutional, Swedish national, and European Union animal care rules and regulations were practiced, and the animal handling permission was acquired from the local Ethics Committee for Animal Research, Uppsala, Sweden (Dnr 5.8.18-06578/2019).

### 4.5. Organ Distribution of [^68^Ga] Ga-NO2A-[Nle^13^]-Col in Mice with Induced Liver Fibrosis

The animals (BALB/c female mice, n = 6) were administered with carbon tetrachloride (CCl_4_) formulated in a corn oil solution (20%, 0.5 mg per gram of body weight) into the lower side of the abdomen three times a week for three weeks. The wellbeing of the animals was monitored for 30 min after the injection and once per day afterwards. The development of liver fibrosis was confirmed by post-mortem Sirius Red staining of the PFA liver biopsies.

Healthy mice (n = 4, 19.6 ± 1.0 g) and mice with CCl_4_ induced liver fibrosis (n = 5, 21.55 ± 1.12 g) were intravenously administered with [^68^Ga]Ga-NO2A-[Nle^13^]-Col (5.2 ± 2.1 MBq, 0.31 ± 0.09 ug and 6.1 ± 2.1 MBq, 0.35 ± 0.08 ug) and the tracer organ distribution was studied after a 60 min incubation ex vivo (Table S1). The weight of the excised organs of interest (blood, heart, lung, liver, pancreas, spleen, kidney, intestines, muscle, brain) was noted and the radioactivity was measured using an automated gamma counter (2480 Wizard2^TM^, PerkinElmer, Waltham, MA, USA) with a correction for dead-time and radioactivity decay.

The organ distribution and kinetics of the uptake was also studied in vivo by the dynamic scanning (60 min) of two healthy mice and two mice with induced fibrosis using a small animal PET-MRI scanner (nanoPET/MRI, Mediso, Medical Imaging Systems, Budapest, Hungary). The PET and MRI imaging data were analyzed using, respectively, PMOD 4.0 (PMOD Technologies, Zurich, Switzerland) and Nucline (Mediso, Hungary) software.

### 4.6. Organ Distribution of [^68^Ga]Ga-NO2A-[Nle^13^]-Col in Healthy Rats and Human Dosimetry Calculation

Sixteen male (n = 10; 363.3 ± 19.6 g) and female (n = 6; 199.2 ± 5.5 g) Sprague–Dawley rats were used for ex vivo organ distribution of the [^68^Ga]Ga-NO2A-[Nle13]-Col for the subsequent extrapolation and calculation of human dosimetry. The male and female animals were administered into the tail vein with, respectively, 2.3 ± 0.8 MBq and 1.4 ± 0.5 MBq, sacrificed at 5, 10, 30, 40, 60, and 120 min post administration, and the organs were harvested, weighted, and measured for their radioactivity content. Samples from the blood, heart, lung, liver, spleen, adrenal glands, kidneys, intestines with or without contents, muscle, testis, bone, brain, pancreas, urine bladder, and bone marrow were collected. To monitor radioactivity elimination and recovery, the radioactivity of the remaining carcass was also measured. The radioactivity measurements were corrected for decay and converted into standardized uptake values (SUVs, Table S2) according to Equation (2), wherein the radioactivity is expressed in MBq and the weight in grams:

(2) $$\mathrm{SUV}=\frac{{\mathrm{Radioactivity}}_{\mathrm{organ}}{*\mathrm{weight}}_{\mathrm{rat}}}{{\mathrm{Radioactivity}}_{\mathrm{injected}}{*\mathrm{weight}}_{\mathrm{organ}}}$$

The rat SUVs were translated into a percentage of injected activity (%IA, Equation (3)) for human organs according to Equation (3) (Table S3), where *SUV_A_* is the SUVs of the rat organs; *g_organ_* and *kg_TBweight_* are, respectively, standard organ weight and standard total body weight of a human. The standard organ masses and total-body weight values were obtained from the OLINDA software (organ level internal dose assessment code, Vanderbilt University, USA, 2007).

(3) $${\left(\%IA_{Organ}\right)}_{human}=SUV_{A}*{\left(\frac{g_{organ}}{kg_{TBweight}}\right)}_{human}$$

The %IA values were decay corrected to the animal termination time point at 5, 10, 30, 40, 60, and 120 min post administration and plotted as a function of time. The area under the curve for each organ was integrated using the trapezoid method assuming a simple exponential decay to eternity after the last point. The derived time-integrated activity coefficient (TIAC) [28] for each organ, which is equal to the number of disintegrations (MBq-h/MBq) and dose rate S-values extracted from the Medical Internal Radionuclide Dose (MIRD) pamphlet [28,29], was used for the computation of organ and whole-body effective doses (mSv/MBq) in humans in the OLINDA/EXM software package (Version 1.1, Vanderbilt University).

### 4.7. Statistical Analysis

The average values (mean) with their corresponding standard deviation (SD) and the significance of difference between groups (Mann–Whitney test) were calculated with Excel (Microsoft) or GraphPad Prism version 7.05 (GraphPad Software, San Diego, CA, USA). The significance level of *p* < 0.05 was applied.

## 5. Conclusions

The improvement of radiolytic stability of the collagelin analogues was achieved by the substitution of methionine with norleucine amino acid residue without a deterioration of the binding capability towards fibrosis. Specific binding to the cryosections of fibrotic mouse liver tissue strongly correlated with the fibrosis score estimated by Sirius Red staining. The blood clearance and tissue washout rates were fast except for the kidneys. With the effective dose being the limiting parameter in both genders, these results would allow for 4–5 PET/CT examinations per year that might be sufficient for longitudinal studies, the monitoring of disease progression and monitoring of treatment response. 

## Figures and Tables

**Table 1:** Estimated absorbed doses (mGy/MBq) of [^68^Ga] Ga-NODAGA-Col, [^68^Ga] Ga-NO2A-Col and [^68^Ga] Ga-NO2A-[Nle^13^]-Col in human females and males extrapolated from rat organ distribution data.

Organ	[^68^Ga] Ga-NODAGA-Col *		[^68^Ga] Ga-NO2A-Col *		[^68^Ga] Ga-NO2A-[Nle^13^]-Col	
	Female	Male	Female	Male	Female	Male
Liver	0.014	0.012	0.007	0.006	0.003	0.002
Spleen	0.011	0.011	0.008	0.006	0.003	0.003
Kidney	0.111	0.100	0.103	0.102	0.135	0.097

* [2].

## Data Availability

Data is contained within the article and Supplementary Materials.

## Supplementary Materials

https://www.mdpi.com/article/10.3390/ph14100990/s1, Table S1. Ex vivo organ distribution of [^68^Ga] Ga-NO2A-[Nle^13^]-Col (SUV) in healthy and induced liver fibrosis BALB/c female mice at 60 min time point.; Table S2. Organ distribution of [^68^Ga] Ga-NO2A-[Nle^13^]-Col (SUV) in Sprague-Dawley rats.; Table S3. Percent of injected activity (%IA) for each entire organ (i.e., %ID/gram multiplied by estimated organ weight) for human male and female extrapolated from rat organ distribution data according to Equation (3) and used as input data for the dosimetry calculations in OLINDA.
