# Supplementary material for: Improved Radiolytic Stability of a 68Ga-labelled Collagelin Analogue for the Imaging of Fibrosis
Source: Pharmaceuticals (Basel). 2021 Sep 28;14(10):990. doi: 10.3390/ph14100990 (PMC8537947; doi:10.3390/ph14100990)
Supplement: Supplementary file 1 [file pharmaceuticals-14-00990-s001.zip › pharmaceuticals-1373066-supplementary.pdf]

pharmaceuticals

MDPI

Research Article/Supplementary

Improved Radiolytic Stability of a ^{68}Ga-labelled Collagelin Analogue for Imaging of Fibrosis

Irina Velikyan [1,2,*], Ulrika Rosenström [3], Maria Rosestedt [1], Olof Eriksson [1] and Gunnar Antoni [1,2,*]

[1] Science for Life Laboratory, Department of Medicinal Chemistry, Uppsala University, SE-75183 Uppsala, Sweden; Maria.rosestedt@ilk.uu.se (M.R.); olof.eriksson@ilk.uu.se (O.E.)
[2] PET-Centre, Centre for Medical Imaging, Uppsala University Hospital, SE-75185, Uppsala, Sweden
[3] Department of Medicinal Chemistry, Uppsala University, SE-75123 Uppsala, Sweden; Ulrika.rosenstrom@ilk.uu.se
* Correspondence: irina.velikyan@akademiska.se (I.V.); gunnar.antoni@akademiska.se (G.A.)

Table S1. Ex vivo organ distribution of [^{68}Ga] Ga-NO2A-[Nle^{13}]-Col (SUV) in healthy and induced liver fibrosis BALB/c female mice at 60 min time point.

**Healthy mice**					
BL	0.1049	0.0563	0.0350	0.0751	
HE	0.0307	0.0248	0.0202	0.0388	
LU	0.0679	0.0438	0.0659	0.0566	
LI	0.0237	0.0219	0.0501	0.0311	
PA	0.0177	0.0116	0.0108	0.0231	
SP	0.0232	0.0195	0.0288	0.0228	
GI	0.0482	0.0767	0.0337	0.1387	
MU	0.0186	0.0048	0.0117	0.0558	
BR	0.0026	0.0022	0.0080	0.0030	
KI	1.6011	1.2802	3.1439	1.8950	
**Mice with induced fibrosis**					
BL	0.3320	0.0285	0.0627	0.0350	0.1061
HE	0.1253	0.0338	0.0279	0.0483	0.1088
LU	0.1791	0.2292	0.0524	0.0631	0.1139
LI	0.1189	0.3567	0.0479	0.0446	0.0520
PA	0.0394	0.0247	0.0323	0.2075	0.0450
SP	0.0969	0.0352	0.0429	0.0935	0.0381
GI	0.0876	0.0186	0.0298	0.0243	0.0515
MU	0.0960	0.0079	0.0363	0.0725	0.0190
BR	0.0273	0.0019	0.0136	0.0110	0.0071
KI	2.1190	2.7798	3.5067	1.8862	2.6319

BL: blood; HE: heart; LU: lungs; LI: liver; PA: pancreas; SP: spleen; GI: gastrointestinal tract; MU: muscle; BR: brain; KI: kidneys.

Table S2. Organ distribution of [^{68}Ga] Ga-NO2A-[Nle^{13}]-Col (SUV) in Sprague-Dawley rats.

Organs	5 min	10 min	30 min	40 min	60 min*	120 min
**MALES**						
BL	2.2227	1.5259	0.7996	0.6235	0.3159	0.0520
HE	0.9703	0.6053	0.3379	0.2248	0.1231	0.0240
LU	1.2793	1.0045	0.5660	0.5628	0.2169	0.0955
LI	0.4781	0.3079	0.1927	0.1977	0.1116	0.0624
SP	0.5412	0.3756	0.2412	0.2053	0.1259	0.0652
PA	0.4725	0.4188	0.2614	0.1995	0.1113	0.0272
AD	0.8204	0.5491	0.4635	0.2843	0.4940	0.2489
KI	19.1672	14.1773	13.6457	13.0941	8.6683	11.4644
INS+	0.7308	0.5005	0.2558	0.4124	0.1324	0.0313
INL-	1.0055	0.9188	0.4759	0.2887	0.2430	0.0668
TE	0.3540	0.3180	0.2161	0.2450	0.1178	0.0322
UB	2.5122	3.1252	5.0138	2.7987	3.7344	0.8958
MU	0.4007	0.5067	0.1163	0.1038	0.1063	0.0164
BO	0.1196	0.2433	0.1431	0.1268	0.2003	0.0384
BM	0.5836	0.5427	0.2621	1.0125	1.1490	0.1543
BR	0.0955	0.0507	0.0346	0.0272	0.0268	0.0058
**FEMALES**						
BL	2.0647	1.4198	0.4969	0.4318	0.2174	0.0412
HE	0.7410	0.6803	0.2069	0.1799	0.1042	0.0260
LU	1.1238	0.9855	0.3847	0.4267	0.2760	0.0631
LI	0.4381	0.4524	0.1706	0.1473	0.1140	0.0889
SP	0.4235	0.3537	0.1737	0.1351	0.0938	0.0660
PA	0.4391	0.3606	0.1636	0.0609	0.0659	0.0290
AD	0.7242	1.2890	0.0127	0.2499	0.1984	0.1276
KI	12.0745	11.3220	6.3247	12.9115	12.8247	9.0780
INS+	0.4361	0.5581	0.2771	0.1648	0.1287	0.0343
INL-	0.5690	0.9104	0.1929	0.2107	0.1035	0.0460
OV	0.9269	1.8741	0.3408	0.3349	0.1587	0.1245
UB	4.8837	6.9611	5.8284	0.8434	1.0761	0.3205
MU	0.5139	0.2950	0.2138	0.0802	0.0488	0.0213
BO	0.3905	0.4033	0.1789	0.1058	0.0640	0.0310
BM	0.4836	0.4427	0.1615	0.7690	0.1130	0.3427
BR	0.0730	0.0964	0.0189	0.0174	0.0149	0.0062

BL: blood; HE: heart; LU: lungs; LI: liver; SP: spleen; PA: pancreas; AD: adrenals; KI: kidneys; INS+: small intestine with its content; INL-: large intestine without content; UB: bladder; GO: gonads; OV: ovarian; MU: muscle; BO: bone; BM: red bone marrow; BR: brain.

*Average of four male rats at 60 min time point.

Table S3. Percent of injected activity (%IA) for each entire organ (i.e. %ID/gram multiplied by estimated organ weight) for human male and female extrapolated from rat organ distribution data according to Equation 3 and used as input data for the dosimetry calculations in OLINDA.

			MALE			
Organs	5 min	10 min	30 min	40 min	60 min	120 min
BL	0.16668	0.11443	0.05997	0.04676	0.02369	0.00390
HE	0.00416	0.00260	0.00145	0.00096	0.00053	0.00010
HE	0.00788	0.00619	0.00349	0.00347	0.00134	0.00059
LU	0.01736	0.01363	0.00768	0.00764	0.00294	0.00130
LI	0.01239	0.00798	0.00499	0.00512	0.00289	0.00162
SP	0.00134	0.00093	0.00060	0.00051	0.00031	0.00016
PA	0.00060	0.00054	0.00033	0.00026	0.00014	0.00003
AD	0.00018	0.00012	0.00010	0.00006	0.00011	0.00006
KI	0.07776	0.05752	0.05536	0.05312	0.03517	0.04651
INS+	0.01091	0.00747	0.00382	0.00615	0.00198	0.00047
INL-	0.00924	0.00844	0.00437	0.00265	0.00223	0.00061
TE	0.00019	0.00017	0.00011	0.00013	0.00006	0.00002
UB	0.00162	0.00202	0.00324	0.00181	0.00241	0.00058
MU	0.15224	0.19252	0.04419	0.03944	0.04040	0.00622
BO	0.00993	0.02021	0.01188	0.01053	0.01664	0.00319
BM	0.00887	0.00825	0.00398	0.01539	0.01746	0.00235
BR	0.00184	0.00098	0.00067	0.00052	0.00052	0.00011
			**FEMALE**			
BL	0.28920	0.15913	0.03230	0.02807	0.01413	0.00268
HE	0.00661	0.00485	0.00086	0.00074	0.00043	0.00011
HE	0.01712	0.01201	0.00272	0.00302	0.00195	0.00045
LU	0.03340	0.02344	0.00531	0.00588	0.00381	0.00087
LI	0.02279	0.01883	0.00412	0.00356	0.00275	0.00215
SP	0.00236	0.00158	0.00045	0.00035	0.00024	0.00017
PA	0.00139	0.00091	0.00024	0.00009	0.00010	0.00004
AD	0.00038	0.00054	0.00000	0.00006	0.00005	0.00003
KI	0.12337	0.09256	0.02999	0.06122	0.06081	0.04304
INS+	0.01580	0.01618	0.00466	0.00277	0.00216	0.00058
INL-	0.01269	0.01624	0.00200	0.00218	0.00107	0.00048
OV	0.00038	0.00061	0.00006	0.00006	0.00003	0.00002
UB	0.05587	0.01810	0.00361	0.00052	0.00067	0.00020
MU	0.32458	0.14908	0.06267	0.02350	0.01429	0.00625
BO	0.06963	0.05755	0.01481	0.00876	0.00530	0.00257
BM	0.10174	0.07315	0.00292	0.01392	0.00205	0.00620
BR	0.00325	0.00344	0.00039	0.00036	0.00031	0.00013

BL: blood; HE: heart; LU: lungs; LI: liver; SP: spleen; PA: pancreas; AD: adrenals; KI: kidneys; INS+: small intestine with its content; INL-: large intestine without content; UB: bladder; GO: gonads; OV: ovarian; MU: muscle; BO: bone; BM: red bone marrow; BR: brain.
